# Drp1 is essential for PINK1/Parkin signaling in H9c2 cardiomyocytes

**DOI:** 10.1002/mco2.231

**Published:** 2023-03-17

**Authors:** Xuecong Ren, Ni Zhang, Xiao‐Yi Chen, Hui Huang, Pei Luo

**Affiliations:** ^1^ Center for Stem Cell and Regenerative Medicine and Zhejiang University Medical Center Zhejiang University School of Medicine Hangzhou China; ^2^ State Key Laboratories for Quality Research in Chinese Medicines Macau University of Science and Technology Macau China; ^3^ Department of Cardiology The Eighth Affiliated Hospital Sun Yat‐sen University Shenzhen China

Dear editor:

Mitochondria are crucial organelles that generate ATP for cell survival. Mitochondria continuously change their morphology via fusion/fission machinery in order to maintain the homeostasis of the mitochondrial population in response to different external and internal stresses via mitochondrial quality control mechanisms. Mitochondrial quality control is mediated by mitophagy pathways that transfer damaged mitochondria to lysosomes for degradation or other secretory/exocytosis pathways that release mitochondria into the extracellular matrix. Three main mitophagy pathways, namely, the PTEN‐induced putative kinase 1 (PINK1)/Parkin signaling pathway, the BCL2 and adenovirus E1B 19‐kDa‐interacting protein 3 (BNIP3) and BNIP3‐like (BNIP3L), also known as NIX, BNIP3/NIX signaling pathway, and the hypoxia‐induced FUN14 domain containing 1 (FUNDC1) signaling pathway, were shown to participate in mitochondrial quality control, especially in neuron cells.^1^ Moreover, mitochondrial‐derived vesicles (MDVs) can selectively bud off damaged mitochondria upon Drp1‐mediated fission and microtubule‐associated motor proteins (MIROs)‐mediated transportation^2^ or this budding can occur independently of fission machinery. In addition, damaged mitochondria can also be transported into migrasomes and subsequently removed from migrating cells.^3^ PINK1/Parkin signaling is activated upon mitochondrial depolarization, which induces PINK1 stabilization on the mitochondrial outer membrane and recruits Parkin to translocate to mitochondria and interact with Mitofusin 2 (Mfn2). Drp1, which is a cytosolic mitochondrial fission regulator that translocates to the mitochondrial outer membrane at mitochondria–endoplasmic reticulum contact site to constrict mitochondria for further segregation, has been found to participate in mitophagy.^4^ However, other studies have reported that Drp1 is involved in Parkin‐independent mitophagy. In primary culture cardiomyocytes and H9c2 cardiac cell line, the relationship between Drp1 and the autophagic process has been studied, but the relationship between Drp1 and PINK1/Parkin signaling is still unknown. In our previous work, we reported that mitochondria in H9c2 cardiomyocytes that become elongated due to D‐galactose exposure contribute to PINK1/Parkin signaling suppression,^5^ which probably occurs due to Drp1 downregulation and dysfunction. Therefore, we wondered whether specific Drp1 deficiency is the crucial factor for the suppression of PINK1 and Parkin mitochondrial translocation in cardiomyocytes.

To address this question, we used small interfering RNA (siRNA) against Drp1 and the Drp1 inhibitor, Mdivi‐1, to downregulate Drp1 expression and suppress its function. We first evaluated the efficient doses of Drp1 siRNA and Mdivi‐1 to determine whether they successfully reduced Drp1 expression in H9c2 cells. Immunoblotting analysis showed that treatment with 100 or 200 nM Drp1 siRNA and 40 μM Mdivi‐1 significantly reduced Drp1 expression (Figure 1A,B and Figure S1A,B). To further confirm the effects of Drp1 siRNA and Mdivi‐1, we analyzed mitochondrial morphology by MitoView Red using confocal microscopy. The confocal images showed that the mitochondrial morphology changed significantly and exhibited an elongated thread‐like pattern after treatment with Drp1 siRNA and Mdivi‐1 (Figure 1C and Figure S1C).

**FIGURE 1:**
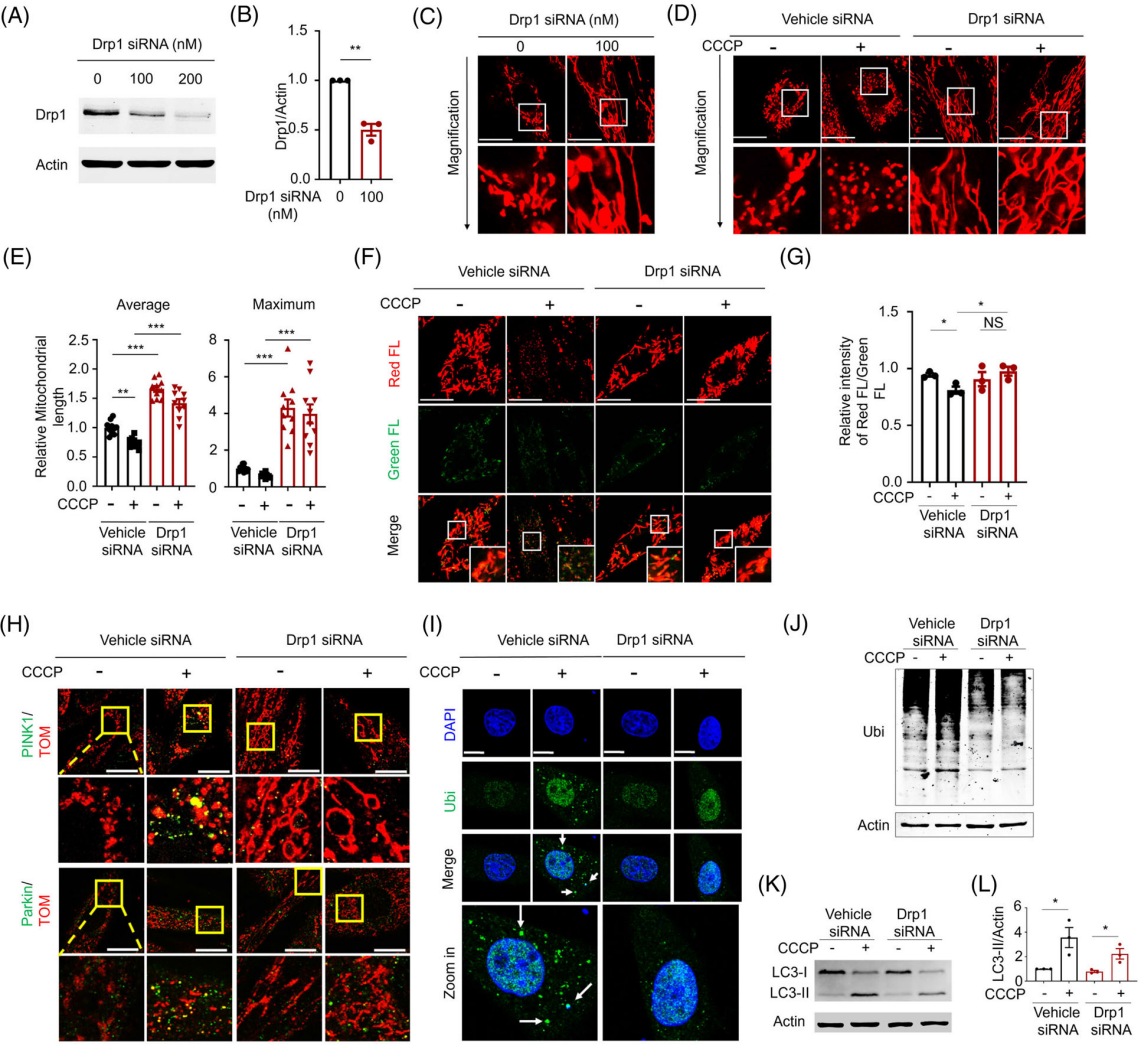
Drp1 is required for CCCP‐induced PINK1/Parkin activation. (A and B) H9c2 cardiomyocytes were treated with Drp1 siRNA (0, 100, 200 nM) for 48 h. Drp1 expressions were detected by immunoblot analysis. Actin was represented as loading control. The relative values were normalized to actin. (C) H9c2 cardiomyocytes were treated with Drp1 (0, 100 nM), and mitochondrial morphology was detected using a confocal microscope by MitoView Red staining (upper panel); objective magnification 63×; white scale bar = 20 μm. Magnified photograph shows a detailed view of the area indicated on the upper panel. (D) CCCP (20 μM) was added to H9c2 cardiomyocytes for 3 h after treating with Drp1 siRNA for 48 h. Mitochondrial morphology was detected using a confocal microscope by MitoView Red staining; objective magnification 63×; white scale bar = 20 μm. Magnified photograph shows a detailed view of the area indicated on the upper panel. Statistical analysis of average, maximum, and minimum length of mitochondria in different groups are shown in (E). (F) CCCP (20 μM) was added to H9c2 cardiomyocytes for 3 h after treating with Drp1 siRNA for 48 h. Mitochondrial membrane potential was detected by using JC‐1 fluorescent dye staining. Images were captured by confocal microscopy to show the variations of red FL; objective magnification 63×; white scale bar = 20 μm. (G) Fluorescent intensity of JC‐1 staining dye was detected by fluorescence microplate assay with Ex: 485/Em: 535 (green FL) and Ex: 550/Em: 600 (red FL). Ratio of red FL to green FL was calculated to show ΔΨm. (H) CCCP (20 μM) was added to H9c2 cardiomyocytes for 3 h after treating with Drp1 siRNA for 48 h. Immunofluorescence analysis of PINK1 and Parkin expressions were detected using confocal microscope; objective magnification 63×; scale bar = 20 μm. Colocalization of PINK1 or Parkin (green) and TOM 20 (red) is shown in magnified images. The yellow fluorescence represents the overlap of red and green fluorescence. (I) Immunofluorescence analysis of poly‐ubiquitin expressions was performed using confocal microscope; objective magnification 63×; scale bar = 10 μm. Poly‐ubiquitin (green) and DAPI (blue) are shown in magnified images. White arrow indicates the accumulation of ubiquitin. (J) CCCP (80 μM) was added to H9c2 cardiomyocytes for 12 h after treating with Drp1 siRNA for 48 h. Poly‐ubiquitin expressions were detected by immunoblot analysis and calculated. Actin was represented as loading control. (K and L) CCCP (80 μM) was added to H9c2 cardiomyocytes for 3 h after treating with Drp1 siRNA for 48 h. LC3 protein expressions were detected by immunoblot analysis. Actin was represented as loading control. The relative values of LC3‐II were normalized to actin. Data (*n* = 3) are shown as the mean ± SEM, *p < 0.05, **p < 0.01, ***p < 0.001, NS: no significance.

Mitochondrial depolarization, which indicates that mitochondria are unhealthy and have lower mitochondrial membrane potential (ΔΨm), is the most important factor that triggers PINK1/Parkin signaling and the mitophagy process. We wondered whether Drp1 downregulation suppressed the decrease in the ΔΨm, which might contribute to the suppression of PINK1/Parkin signaling. Thus, we used carbonyl cyanide 3‐chlorophenylhydrazone (CCCP), which is a mitochondrial electron transport chain uncoupler, that contributes to mitochondrial depolarization and fragmentation. We first analyzed mitochondrial morphology after CCCP treatment, and confocal images showed that CCCP treatment significantly changed the mitochondrial morphology into a punctate‐like structure, compared with the groups without CCCP treatment (Figure 1D and Figure S1D). However, when cells were preincubated with Drp1 siRNA and Mdivi‐1, the effect of CCCP on inducing mitochondrial morphological changes was blocked (Figure 1D and Figure S1D). Further statistical analysis showed that CCCP could reduce mitochondrial lengths in control cells, but not in cells treated with Drp1 siRNA and Mdivi‐1 (Figure 1E and Figure S1E). Next, we evaluated whether Drp1 downregulation would also block the decrease in the ΔΨm under CCCP treatment conditions by using 5,5′,6,6′‐tetrachloro‐1,1′,3,3′‐tetraethyl‐imidacarbocyanine iodide (JC‐1) staining dye. In the control groups, CCCP decreased the intensity of aggregated form of JC‐1 (indicated by red fluorescence). However, in the Drp1 siRNA and Mdivi‐1 groups, the red fluorescence intensity was not changed after CCCP treatment (Figure 1F and Figure S1F). The ΔΨm was statistically analyzed by calculating the ratio of red fluorescence to green fluorescence of JC‐1 staining dye, which showed CCCP reduced ΔΨm in control groups but not in the Drp1 siRNA and Mdivi‐1 groups, consistent with the observations of fluorescent images (Figure 1G and Figure S1G). Taken together, these results show that Drp1 downregulation blocked mitochondrial fragmentation and depolarization under CCCP treatment in H9c2 cardiomyocytes.

Previously, we found that mitochondrial elongation ameliorated PINK1/Parkin signaling activation, but several mitochondrial membrane proteins, such as mitochondrial adaptor fission 1 (Fis1), mitochondrial fission factor (MFF), and even other proteins located on the ER or Golgi, also participated in the process of mitochondrial fission. Therefore, we were not certain whether Drp1 was the most crucial molecule for regulating PINK1/Parkin signaling in cardiomyocytes. Based on this, we first induced the mitochondrial translocation of PINK1 and Parkin under CCCP treatment condition. The confocal images showed that CCCP prominently induced the colocalization of TOM20 (mitochondrial outer membrane protein, red fluorescence [FL]) and PINK1 (green FL), as well as Parkin (green FL), indicating mitochondrial translocation of PINK1 and Parkin. However, this phenomenon was blocked in Drp1‐knockdown cells, suggesting that Drp1 was required for the mitochondrial translocation of PINK1 and Parkin (Figure 1H and Figure S1H).

The PINK1/Parkin‐mediated mitophagy process is followed by autophagic progression under conditions of Parkin‐mediated mitochondrial proteins’ ubiquitination. We further evaluated ubiquitination levels under long‐term (12 h) CCCP treatment condition. The green fluorescence indicating ubiquitinated proteins was dramatically increased. However, in the Drp1 knockdown cells, green fluorescent puncta were barely observed (Figure 1I and S1I). Consistently, the CCCP‐induced ubiquitinated protein levels were measured by immunoblotting analysis, and the results showed that Drp1 downregulation suppressed CCCP‐induced protein ubiquitination (Figure 1J and Figure S1J). Moreover, mitochondrial proteins are transferred into autophagosomes after Parkin (E3 ubiquitin ligase)‐mediated ubiquitination. Therefore, we further measured the expression of the autophagic marker LC3. Immunoblotting and statistical analysis showed that the increasing level of LC3‐II induced by CCCP treatment was reduced under Drp1 siRNA and Mdivi‐1 treatment (Figure 1K,L and Figure S1K,L). These results demonstrated that Drp1 was essential for PINK1/Parkin‐activated mitochondrial autophagic and ubiquitinated processes.

In summary, Drp1 downregulation induced mitochondrial elongation, and elongated mitochondria were more resistant to CCCP‐induced mitochondrial fragmentation and depolarization; thus, Drp1 downregulation contributed to the suppression of PINK1 and Parkin mitochondrial translocation in H9c2 cardiomyocytes. As Parkin is an E3 ubiquitin ligase, Drp1 downregulation further suppressed mitochondrial protein ubiquitination and subsequent autophagic activation. Collectively, Drp1 was essential for PINK1/Parkin signaling activation under mitochondrial depolarization conditions in H9c2 cardiomyocytes. H9c2 cardiomyocytes cell line is originated from rat heart tissue that exhibits many of the properties of skeletal muscle and representatively used in cardiovascular disease research. We previously demonstrated that senescent H9c2 cells displayed elongated mitochondria mediated by fission, not fusion, machinery. This mitochondrial elongation induced by fission deficiency suppressed mitochondrial depolarization and the subsequent PINK1/Parkin‐mediated mitophagy process. Here, we showed supplementary evidence to confirm the dominant role of Drp1 in regulating PINK1/Parkin signaling, which probably explains the reduced PINK1/Parkin signaling in senescent H9c2 cells. Although we demonstrated the crucial function of cardiac Drp1 in PINK1/Parkin signaling, the exact relationship between Drp1 and PINK1/Parkin and how Drp1 regulates the mitochondrial translocation of PINK1 and Parkin remain unknown, even if PINK1 can phosphorylate Drp1 to promote mitochondrial fission independent of mitophagy. Further specific mechanical studies of Drp1 and its cofactor in regulating PINK1/Parkin signaling should be performed in cardiovascular system research.

## AUTHOR CONTRIBUTIONS

Conceptualization: Pei Luo and Xuecong Ren. Methodology and data analysis: Xuecong Ren, Ni Zhang, Xiao‐Yi Chen, and Hui Huang. Writing original draft: Xuecong Ren. Supervision and project administration: Pei Luo. All authors have read and approved the final manuscript.

## CONFLICT OF INTEREST STATEMENT

The authors declare no conflicts of interest.

## FUNDING STATEMENT

This work was supported by Macao Science and Technology Development Fund (0028/2019/AG), National Nature Science Foundation of China (31900498) and Postdoctoral Science Foundation of China (2019M662037).

## ETHICS STATEMENT

No ethical approval was necessary for this work.

## Supporting information

Supporting Information

## Data Availability

The datasets generated during and/or analyzed during the current study are available from the corresponding author on reasonable request.
