# Supplementary material for: Drp1 is essential for PINK1/Parkin signaling in H9c2 cardiomyocytes
Source: MedComm (2020). 2023 Mar 17;4(2):e231. doi: 10.1002/mco2.231 (PMC10022580; doi:10.1002/mco2.231)
Supplement: Supplementary file 1 — Supporting Information [file MCO2-4-e231-s001.docx]

**Supplementary Information**

**Drp1 is essential for PINK1/Parkin signaling in H9c2 cardiomyocytes**

Xuecong Ren^1,2†^, Ni Zhang^1†^, Xiao-Yi Chen^2^, Hui Huang^3^, Pei Luo^2^*

^1^Center for Stem Cell and Regenerative Medicine and Zhejiang University Medical Center, Zhejiang University School of Medicine, Hangzhou 310058, China

^2^State Key Laboratories for Quality Research in Chinese Medicines, Macau University of Science and Technology, Macau, China

^3^Department of Cardiology, The Eighth Affiliated Hospital, Sun Yat-sen University, Shenzhen 518048, China

†These authors contributed equally

* Correspondence to: Dr. Pei Luo, E-mail: pluo@must.edu.mo

**Materials and methods**

**Cell culture and transfection**

Rat H9c2 cardiomyocyte (CRL1446, ATCC, USA) was cultured in Dulbecco’s Modified Eagle’s Medium (DMEM, Gibco, Oklahoma, USA) supplemented with 10% fetal bovine serum (FBS, Gibco, Oklahoma, USA) and 1% v/v penicillin/streptomycin (Gibco, Oklahoma, USA) at 37°C in a 5% CO_2_ humidity environment. H9c2 cells were transfected by Non-Targeting control (D-001810-10-05, Dharmacon) and Drp1 siRNA (L-088074-02-0005, Dharmacon) with lipofectamine 3000 (Thermo Fisher, USA) for 48 hours.

**Pharmacological treatments**

Carbonyl cyanide 3-chlorophenylhydrazone (CCCP, Sigma, USA) and Mitochondrial division inhibitor 1 (Mdivi-1, Sigma, USA) were dissolved in dimethylsulfoxide (DMSO, ACROS, USA).

**Evaluation of mitochondrial morphology**

H9c2 cells were seeded into μ-Slide 8-well glass bottom plate (#80826, ibidi, Germany). After transfection by Drp1 siRNA and treatments of Mdivi-1 (40 μM, Sigma, USA), the cells were incubated with 50 nM MitoView Red (GeneCopoeia, USA) at 37 ˚C for 30 min. Mitochondrial morphology in each group was captured using a confocal microscope (LEICA TCS SP8, Germany) equipped with 63 × oil immersion objective. Red fluorescence represents mitochondria.

**Mitochondrial membrane potential (ΔΨm) determination**

JC-1 mitochondrial membrane potential assay kit (Abcam, Cambridge, UK) was used to measure the ΔΨm. Fluorescent images were captured using a confocal microscopy to indicate JC-1 staining. For the quantification of ΔΨm, H9c2 cells were seeded in black plate and Ex 488/Em 530 nm and Ex 550/Em 600 nm were used to measure the JC-1 fluorescence and the ratio of red to green fluorescence was calculated.

**Immunoblot analysis**

Cells and were lysed with RIPA buffer (20 mM Tris-HCl (pH 7.5), 150 mM NaCl, 1 mM Na_2_EDTA, 1 mM EGTA, 1% NP-40, 1% sodium deoxycholate, 2.5 mM sodium pyrophosphate, 1 mM beta-glycerophosphate, 1 mM Na_3_VO_4_, 1 µg/ml leupeptin, Cell Signaling Technology, USA) containing protease and phosphatase inhibitors (Roche, Basel, Switzerland) on ice. Samples were centrifuged at 13000 rpm at 4 ˚C for 10 min, and the supernatant was collected to a new and clear tube. Equal amounts of proteins were boiled and separated with 8-12% SDS-PAGE gels and transferred to a nitrocellulose membrane (Millipore, Germany). After blocking, the membrane was incubated overnight at 4 ˚C with primary antibodies of anti-Drp1 (1:1000, Cell Signaling Technology, USA), anti-LC3 (1;1000, Cell Signaling Technology, USA), anti-ubiquitin (1:500, Abcam, UK). Subsequently, membranes were incubated with secondary antibody at room temperature. Actin (1:10000, Sigma, USA) was used as a loading control.

**Immunocytochemistry analysis**

H9c2 cells were seeded into μ-Slide 8-well glass bottom plate at a total number of 7500 per well. After treatment, cells were washed with PBST (0.1% Tween 20 to PBS) and fixed with 4% PFA (15min, RT), then permeabilized with 0.1% Triton X-100 (10min, RT). The cells were washed with PBST for 3 times and blocked with 1% BSA/PBST for 1 hour at room temperature, incubated with primary antibody (TOM20 1:50, Abcam; Parkin 1:200, Abcam; PINK1 1:200, Novus; Ubiquitin 1:100, Abcam) overnight in 4 ˚C. Secondary was used in 1:250 in room temperature for 2 hours, respectively. Fluorescent image was detected using a confocal microscope equipped with 63 × oil immersion objective.

**Statistical Analysis**

Data were analyzed using GraphPad Prism 9.0 (GraphPad Software Inc., USA), and all results were expressed as means ± SEM. Student’s t-test was used to analyze difference between two groups. Values with *p* < 0.05 were considered statistically significant.


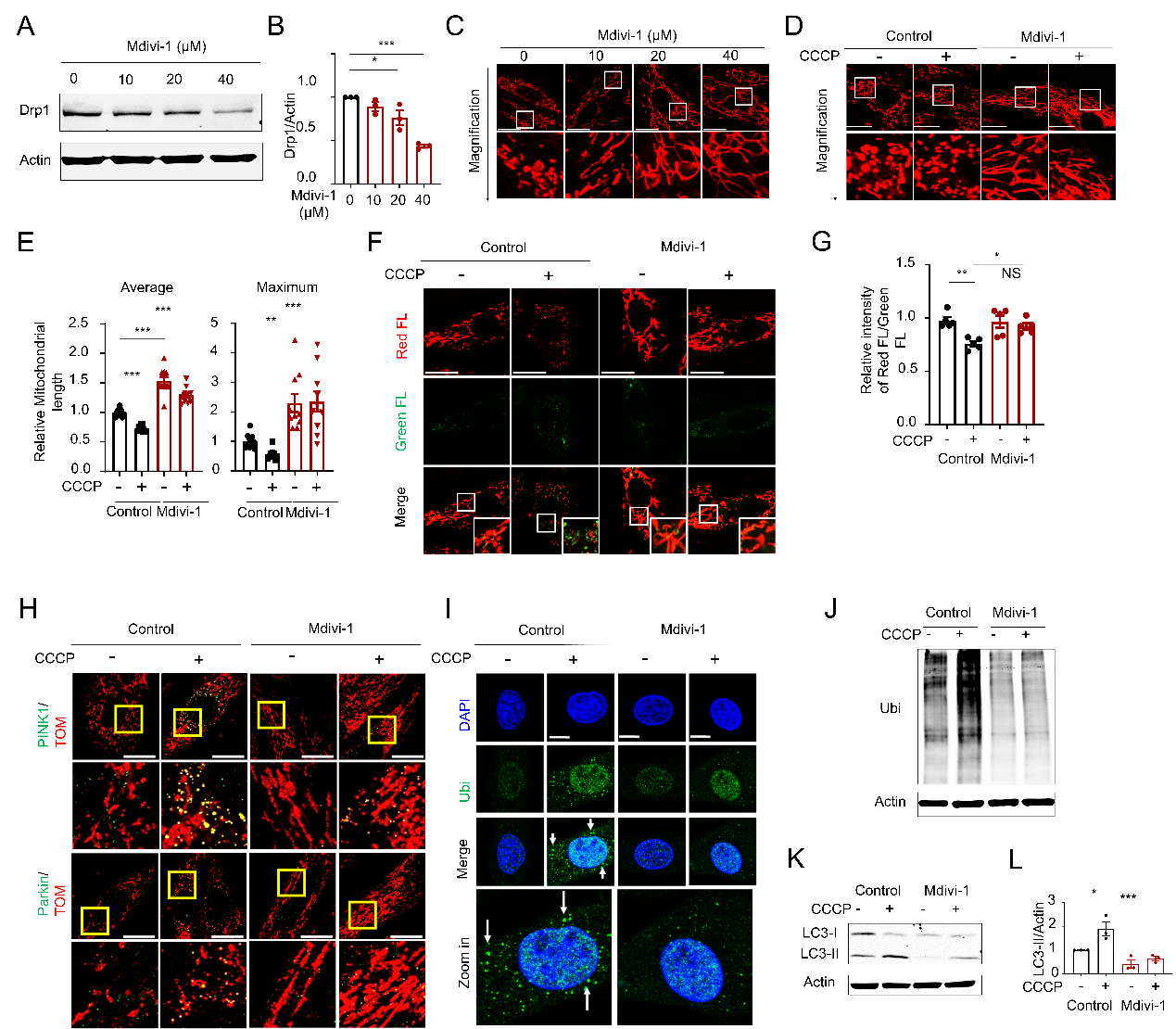


**Supplementary Fig 1. Mdivi-1-mediated Drp1 downregulation suppressed PINK1/Parkin signaling.**

**(A, B)** H9c2 cardiomyocytes were treated with Mdivi-1 (0, 10, 20, 40 μM) for 48 hours. Drp1 expressions were detected by immunoblot analysis. Actin was represented as loading control. The relative values were normalized to Actin. **(C)** H9c2 cardiomyocytes were treated with Mdivi-1 (0, 10, 20, 40 μM) and mitochondrial morphology was detected using a confocal microscope by MitoView Red staining (upper panel); objective magnification 63×; white scale bar represents 20 μm. Magnified photograph showed a detail view of the area indicated on the upper panel. **(D)** CCCP (20 μM) were added to H9c2 cardiomyocytes for 3 hours after treating with Mdivi-1 for 48 hours. Mitochondrial morphology was detected using a confocal microscope by MitoView Red staining; objective magnification 63×; white scale bar represents 20 μm. Magnified photograph showed a detail view of the area indicated on the upper panel. Statistical analysis of average, maximum and minimum length of mitochondria in different groups were shown in **(E)**. **(F)** CCCP (20 μM) were added to H9c2 cardiomyocytes for 3 hours after treating with Mdivi-1 for 48 hours. Mitochondrial membrane potential was detected by using JC-1 fluorescent dye staining. Images were captured by confocal microscope to show the variations of red and green FL; objective magnification 63×; white scale bar represented 20 μm. **(G)** Ratio of red/green FL was calculated by fluorescence microplate assay. **(H)** CCCP (20 μM) were added to H9c2 cardiomyocytes for 3 hours after treating with Mdivi-1 for 48 hours. Immunofluorescence analysis of PINK1 and Parkin expressions were detected using confocal microscope; objective magnification, 63×; scale bar represents 20 μm. Colocalization of PINK1 or Parkin (green) and TOM 20 (red) were shown in magnified images. The yellow fluorescence represented the overlap of red and green fluorescence. **(I)** Immunofluorescence analysis of poly-ubiquitin expressions were detected using confocal microscope; objective magnification, 63×; scale bar represents 10 μm. Poly-ubiquitin (green) and DAPI (blue) were shown in magnified images. White arrow indicated the accumulation of ubiquitin. **(J)** CCCP (80 μM) were added to H9c2 cardiomyocytes for 12 hours after treating with Mdivi-1 for 48 hours. Poly-ubiquitin expressions were detected by immunoblot analysis and calculated. Actin was represented as loading control. **(K, L)** CCCP (80 μM) were added to H9c2 cardiomyocytes for 3 hours after treating with Mdivi-1 for 48 hours. LC3 protein expressions were detected by immunoblot analysis. Actin was represented as loading control. The relative values of LC3-II were normalized to Actin. Data (n=3) were shown as the mean ± SEM, **p*<0.05, ***p*<0.01, ****p*<0.001.
